# Dutch General Practitioners’ weight management policy for overweight and obese patients

**DOI:** 10.1186/2052-9538-1-2

**Published:** 2014-02-19

**Authors:** Corelien JJ Kloek, Jacqueline Tol, Cindy Veenhof, Ineke van der Wulp, Ilse CS Swinkels

**Affiliations:** NIVEL Utrecht, PO Box 1568, 3500 BN Utrecht, The Netherlands; VU University Medical Center Amsterdam, Amsterdam, The Netherlands

**Keywords:** Obesity, General practitioners, Public health, Referral and consultation

## Abstract

**Background:**

General practitioners (GPs) can play an important role in both the prevention and management of overweight and obesity. Current general practice guidelines in the Netherlands allow room for GPs to execute their own weight management policy.

**Objective:**

To examine GPs’ current weight management policy and the factors associated with this policy.

**Methods:**

800 Dutch GPs were asked to complete a questionnaire in December 2012. The questionnaire items were based on the Dutch Obesity Standard for GPs. The data were analyzed by means of descriptive statistics and multiple linear regression analyses in 2013.

**Results:**

In total, 307 GPs (39.0%) responded. Most respondents (82.9%) considered weight management as part of their responsibility for providing care. GPs aged <48 years discussed weight less frequent. Next, weight is less frequently discussed with patients without weight-related comorbidities or with moderately overweight patients compared to obese patients. On average, 47.7% of the GPs reported to refer obese patients to a weight management professional, preferably a dietitian (98.3%). GPs with a BMI ≥ 25 kg/m^2^ were less likely to refer obese patients. In addition, GPs who had frequent contact with a dietitian were more likely to refer obese patients.

**Conclusions:**

In the context of General Practice and preventive medicine, GPs’ discussion of weight and the variety of obesity-determinants with their moderately overweight patients deserves more attention, especially from younger GPs. Strengthening interdisciplinary collaboration between GPs and dietitians could increase the referral percentage for dietary treatment.

**Electronic supplementary material:**

The online version of this article (doi:10.1186/2052-9538-1-2) contains supplementary material, which is available to authorized users.

## Background

Overweight and obesity constitute a global problem, denoted by the World Health Organization (WHO) as “globesity”. In 2008, 35% of the adults worldwide were overweight, defined as a Body Mass Index (BMI) of 25.0-30.0 kg/m^2^ and additionally 12% of the adults were obese, defined as a BMI ≥ 30.0 kg/m^2^ [1]. The number of people with overweight and obesity has increased rapidly in recent decades. In the Netherlands alone, the prevalence of adults with overweight increased from 28.2% to 36.8% between 1981 and 2011. In addition, the prevalence of obesity doubled from 5.3% to 11.4% during the same period [2]. Without preventive action it is estimated that overweight and obesity in the Netherlands may affect two thirds of the adult population by 2024 [3]. As a consequence, Dutch healthcare costs directly related to overweight and obesity are substantial [4]. Overweight and obesity are important risk factors for chronic diseases like Type 2 Diabetes Mellitus, cardiovascular diseases, different types of cancer (endometrial, breast, colon) osteoarthritis [5] and are related to poorer quality of life [6]. In addition, obesity is significantly associated with major depressive disorders and anxiety disorders [7].

As gatekeepers in the Dutch health care system, General Practitioners (GPs) can play an important role in both the prevention and management of overweight and obesity. Nearly 80% of all Dutch citizens visit their GP at least once a year [8]. People with obesity consult their GP more often than those without obesity [9]. Guidelines for GPs’ weight management policy are outlined in the Obesity Standard of the Dutch College of General Practitioners (NHG). Diagnostics and treatment are indicated for patients with a BMI ≥ 25.0 kg/m^2^, weight-related comorbidities or increased cardiovascular risks. For overweight patients with an excessive waist circumference, diagnostics and treatment are only indicated if weight is the patient’s reason for consultation. Treatment may consist of counseling about nutrition, physical exercise, motivation, and discussion about environmental influences, psychosocial problems and weight-related health risks [10]. Referral to other health care providers (i.e. dietitian or nurse practitioner) is indicated in the following situations: if requested by the patient, if underlying causes such as psychological problems are suspected, if previous attempts to lose weight have failed or if the patient needs comprehensive support [10].

Previous studies have shown that GPs intervene in terms of diagnostics and treatment in only half of the patients with obesity, but specific information about The Netherlands is missing [11, 12]. Barriers among GPs to discussing weight with their patients were a lack of time, insufficient knowledge, inadequate skills, lack of confidence and insufficient motivation among patients [13–20]. Female doctors were more likely to deliver weight-related counseling and were more prevention orientated in obesity management compared to their male colleagues [21]. Also GPs’ age has been shown to be related to their attitude regarding weight management policy, although results are inconsistent [22, 23]. Finally, GPs who themselves were conscious of their personal diet, appeared to calculate patients’ BMI more frequently [14].

With regard to the referral percentage of patients with obesity for nutrition and/or dietary advice, previous studies have reported a relationship with GPs’ attitude toward other health care providers. Mathus-Vliegen et al. reported that because of some GPs’ negative attitude to dietitians, they often do not refer patients with obesity to these health care providers [24]. Moreover, problems with interdisciplinary communication impede GPs from referring overweight and obese patients [17, 24, 25]. Costs involved with dietary treatment were cited as a further inhibiting factor in referring patients to other health care providers for nutrition and/or dietary advice [26].

Clearly, the increasing prevalence and the seriousness of overweight and obesity highlight the necessity for solutions. Because of their central role in primary care, GPs are regarded as the principal health care providers in the management of overweight and obesity. Although guidelines for weight management are contained in the NHG Obesity Standard, there is a lack of information about GPs’ weight management policy in daily practice. Multiple factors have been found to be associated with GPs’ weight management policy, however there may be other influencing factors related to the GP, which may be informative in improving weight related referral rates. New in this study is the combination of the survey of GPs’ current weight management policy and the analysis of the factors associated with this policy. The objective of the present study is to explore GPs’ policy on the management of overweight and obesity as well as factors associated with this policy.

## Methods

### Design and study population

This study was conducted in a cross-sectional design. A random sample of 800 registered Dutch GPs representative of gender, age, type of employment, type of practice and degree of urbanicity were invited to participate. The GPs were recruited from the national register database for primary health care providers of the Dutch Institute for Health Services Research (NIVEL) [27]. Those working as temporary employees were excluded from the study. According to the Dutch Medical Research Involving Human Subjects Act this study did not require ethics approval.

### Data collection

The data were collected by means of a questionnaire measuring GPs’ weight management policy. For the purpose of this study several questions were developed and included in a larger postal survey. The complete questionnaire included 26 questions (12 were used for the current study, see Additional file 1). The 26 questionnaire items (Additional file 1) were based on the National Obesity Standard for GPs [10]. The items were measured on either a ratio, ordinal or nominal level. The questionnaire comprised two sections of which the first included general questions with regard to overweight and obesity. The second section focused on patients with obesity solely, because an intervention is always indicated for these patients [10]. Nine researchers provided reviews on the scope, length and comprehensibility of the questionnaire. After these expert evaluations, minor modifications were made. The questionnaire was sent by post in December 2012 and took approximately 10 minutes to complete. A reminder was sent in January 2013.

### Statistical analysis

Data analysis was performed using Stata version 12 (StataCorp LP, College Station, Texas, USA) in 2013. The results were processed anonymously. Based on the NIVEL database, general details were available on the GPs who did not participate. Non-response analyses were performed by using t-tests and Chi-squared tests. Missing values were excluded in the analyses. The answer “do not know” was treated as a missing value. Assumptions of statistical techniques were checked.

GPs’ policy on managing overweight and obesity was determined by means of descriptive statistics on questionnaire items 1, 2, 3, 4 and 10 (Additional file 1).

The frequency of discussing weight was determined by adding up the respondents’ answers on item 2 (7 sub-items, 4-point scaled) of the questionnaire. The possibility of merging these 7 different sub-items was investigated by using the Spearman correlation test and a calculated Cronbach’s alpha. Items with −0.80 < r < 0.80 were merged, as these item associations were considered (fairly) strong. Likewise, a Cronbach’s alpha score of >0.70 was considered as good internal consistency [28]. The generated sum score for “discussing weight” ranged from 7 to 28 points, where higher scores indicated that GPs more often discussed weight with their patients.

Characteristics associated with GPs’ policy, i.e. the dependent variables, discussing weight sum score and referral percentage for nutrition and/or dietary advice, were analyzed univariately in separate analyses, by means of t-tests and Chi-squared tests. Independent variables were GPs’ gender, age, type of employment, BMI, vision about duties of care, perception of other health care providers’ suitability for weight management, frequency of contact with a dietitian, type of practice and degree of urbanicity. Independent variables with p < 0.15 in univariate analyses were included in a multiple linear regression model. In the case of absence of linearity between the independent and dependent variables, 5-point scaled items were transformed into 3-point scaled items. In the case of linearity, continuous variables were centered to the mean for better interpretation. In multivariable analysis a, p < 0.05 was considered as statistically significant.

## Results

### Respondents and non-respondents

Of the 800 questionnaires distributed, 12 were returned because of incorrect addressing or because the GPs appeared to be retired. From the final sample of 788 GPs, the net response rate was 39.0% (N = 307). Table 1 presents the characteristics of those who participated in the survey compared to the non-respondents. Non-response analyses showed no statistically significant differences between respondents and non-respondents. Table 2 shows the results of the questionnaire. On average, GPs’ BMI appeared to be 23.5 kg/m^2^ (SD 2.6; min-max: 17.4-31.7 kg/m^2^). Nearly a quarter (24.9%) of the GPs were overweight.

**Table 1:** **General characteristics of respondents and non-respondents**

Variable	Respondents n = 307 (39.0%)	Non-respondents n = 481 (61.0%)	p-value non-response analysis
**Gender** ^**a**^ **:**			
Male	142 (51.4%)	246 (51.1%)	0.93
Female	134 (48,6%)	235 (48.9%)	
**Age**, mean	48.3 (SD 9.2)	47.9 (SD 8.8)	0.63
<40	71 (23.1%)	100 (20.8%)	
40-49	91 (29.7%)	164 (34.1%)	
≥50	145 (47.2%)	217 (45.1%)	
**Type of employment** ^**a**^ **:**			
Private	226 (81.9%)	416 (86.5%)	0.09
Salaried	50 (18.1%)	65 (13.5%)	
**Type of practice:**			
Solo	60 (19.5%)	90 (18.7%)	0.45
Dual	119 (38.8%)	208 (43.2%)	
Group	128 (41.7%)	183 (38.1%)	
**Urbanicity** ^**b**^ **:**			
Urban	158 (51.5%)	158 (46.4%)	0.31
Suburban	56 (18.2%)	56 (18.5%)	
Rural	93 (30.3%)	93 (35.1%)	

^a^N = 276 Respondents.

^b^Urbanicity: Urban: ≥1500 addresses per km^2^/Suburban: 1000–1499 addresses per km^2^/Rural: <1000 addresses per km^2^.

**Table 2:** **General results from questionnaire**

Variable	N^c^	%
**GPs’ BMI:**		
<25	217	75.1
≥25	72	24.9
**Frequent contact with a dietitian:**		
No	164	54.0
Yes	140	46.0
**Specialized health care providers in building** ^**a**^ **:**		
No	50	16.3
Yes	257	83.7
**Dietitian in building:**		
No	166	54.1
Yes	141	45.9
**GPs’ perception of health care provider suitability** ^**b**^ **:**		
**-GP:**		
Not at all/somewhat	201	67.2
Mainly/very suitable	98	32.8
**-Nurse practitioner:**		
Not at all/somewhat	87	29.1
Mainly/very suitable	212	70.9
**-Dietitian:**		
Not at all/somewhat	5	1.7
Mainly/very suitable	295	98.3
**-Weight-management consultant:**		
Not at all/somewhat	27	11.7
Mainly/very suitable	204	88.3
**-Psychologist:**		
Not at all/somewhat	189	65.4
Mainly/very suitable	100	34.6
**-Physical therapist:**		
Not at all/somewhat	184	63.2
Mainly/very suitable	107	36.8

^a^Health care providers who deliver nutritional and/or dietary advice.

^b^Suitability of providing weight management for obese patients.

^c^Due to missing values, N differs per question.

### GPs’ vision and frequency of discussing weight

**Figure 1:**
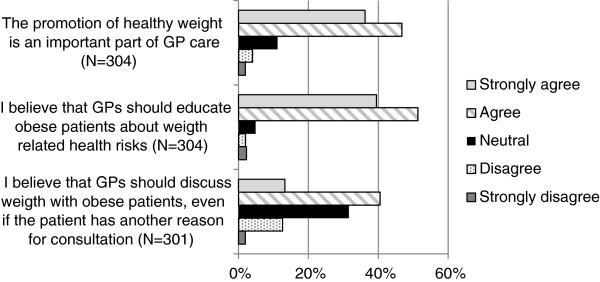
**GPs’ vision about weight management as part of GP care.**

**Figure 2:**
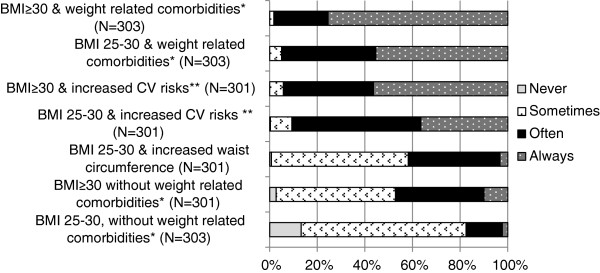
**Frequency of discussing weight during consultations, for different populations.** *For example osteoarthritis, DMII. **For example familial cardiovascular diseases, high blood pressure.

Figure 1 shows GPs’ perception about overweight and obesity management. Most respondents (82.9%) agreed that promoting a healthy weight is an important part of GP care. Likewise, a majority (90.8%) agreed that GPs should educate patients with obesity about potential health risks. A smaller percentage (53.8%) agreed that GPs should discuss weight, even if the obese patient has another reason for the consultation. Figure 2 shows GPs’ reported frequency of discussing weight for different stages of overweight and obesity. GPs were less likely to discuss weight with patients who had lower BMI and/or no weight-related health risks.

### Weight-related topics discussed by the GP

Weight-related topics that were most frequently broached by GPs during consultations were patients’ motivation for weight loss (84.0%), amount of physical exercise (81.4%), weight-related health risks (77.5%), nutrition pattern (72.3%) and weight loss efforts in the past (67.1%). Additionally, 57.7% of the GPs reported to discuss possible interventions to achieve weight loss. Less often discussed by GPs were patients’ current medication use (21.5%), psychosocial problems (32.3%) and environmental influences on weight (35.5%).

The two most frequently reported reasons for not talking about weight with an obese patient were “already talked about weight in the past” (76.9%) or “not having enough time” (59.9%). Patients lacking motivation (24.4%) and “afraid to negatively influence the relationship with the patient” (23.1%) were less frequently cited.

### Factors associated with frequency of discussing weight

The mean score for GPs discussing weight sum score was 21.1 (SD 2.7; min-max: 14–28). In univariate analyses, GPs’ age and their vision about promoting a healthy weight as an important part of GP care, were associated (p < 0.15) with the discussing weight sum score. Table 3 shows the results from the multiple linear regression model. The discussing weight sum score increased by 0.06 points for every year that the GP’s age was above the mean of 48 years. Further, GP’s vision about promoting a healthy weight as an important part of GP care, was related to the frequency of discussing weight. The discussing weight sum score increased by 0.71 points for every point (on a 5-point scale) that GPs agreed more with the assertion that promoting a healthy weight is an important part of GP care.

**Table 3:** **Multiple regression model of GP-related factors associated with discussing weight (scale 7–28) (N = 303)**

Variable	Coefficient (95% C.I.)	p-value
Age (mean):	0.06 (0.03; 0.10)	<0.01
Vision about GPs’ duties of care:		
-Promoting healthy weight as an important part of GP care:	0.71 (0.37; 1.05)	<0.01
Intercept	18.13 (16.70; 19.56)	

### Collaboration with other health care providers for weight management

Most GPs (83.7%) reported the presence of one or more health care providers specialized in nutrition and/or dietary advice in the same medical center. Nearly half (46.0%) reported having frequent contact with a dietitian. The majority (98.3%) regarded the dietitian as a suitable health care provider for the dietary treatment of patients with obesity. Nearly a third (32.8%) of GPs regarded themselves as a suitable health care provider for obesity treatment (Table 2). Most frequently reported reasons for not referring to a dietitian were lack of patients’ motivation for weight loss (63.8%), the fact that patients did not want to receive help from a dietitian (54.7%) and high costs of dietitian consultations (38.1%).

### Referral percentage for obesity management and associated factors

GPs’ average self-reported referral percentage of patients with obesity to other health care providers for nutrition and/or dietary advice was 47.7% (SD 27.8).

Univariate analyses showed that GPs’ BMI, frequent contact with a dietitian, the presence of a dietitian in the same medical building, vision of educating patients with obesity about weight-related comorbidities and discussing weight with patients were associated with the referral percentage for obesity management (p < 0.15).

Table 4 shows the results from the multiple linear regression model. Overweight or obese GPs (BMI ≥ 25) were significantly related to an 11.6% lower referral percentage for obesity management compared to those with a healthy weight. GPs’frequently in contact with a dietitian were significantly related to an increase of 11.8% in referral rate compared to GPs who were not frequently in contact with a dietitian. GPs who agreed with the assertion that educating patients with obesity about weight-related comorbidities is part of GP care reported a significantly higher referral percentage (24.1%) for obesity management.

**Table 4:** **GP-related factors associated with referrals for obesity management (N = 248)**

Variable	Coefficient (95% C.I.)	p-value
**GPs’ BMI:**		
<25	*Reference*	*Reference*
≥25	−11.6 (−19.5; −3.7)	<0.01
**Frequent contact with a dietitian:**		
No	*Reference*	*Reference*
Yes	11.8 (4.2; 19.3)	<0.01
**Dietitian in building:**		
No	*Reference*	*Reference*
Yes	0.9 (−6.6; 8.5)	0.81
**Vision about GPs’ duties of care: -Educating patients with obesity about weight-related comorbidities:**		
Disagree	*Reference*	*Reference*
Neutral	17.5 (−8.9; 44.0)	0.19
Agree	24.1 (4.2; 44.0)	0.02
**Discussing weight:**		
Score 7−18	*Reference*	*Referencesss*
Score 19−20	−4.7 (−16.1; 6.6)	0.41
Score 21−22	0.1 (−10.6; 10.8)	0.98
Score 23−28	10.5 (−0.5; 21.4)	0.06
**Intercept**	**19.6 (−2.3; 41.5)**	

## Discussion and conclusion

This explorative study showed that most GPs (82.9%) considered weight management for overweight and obese patients as part of their responsibility for providing care. However, weight is less frequently discussed by younger GPs. Next, weight is less frequently discussed with patients without weight-related comorbidities or with moderately overweight patients compared to obese patients. Nearly half of the GPs reported to refer obese patients to a weight management professional, preferably a dietitian. In addition, GPs who had frequent contact with a dietitian and those who felt more responsible for educating patients with obesity about weight-related comorbidities were more likely to refer obese patients. Finally, overweight and obese GPs were less likely to refer obese patients. The results of this study may be used to improve consistency in GPs’ weight management policy, for example, by means of communication and education materials.

This paper identified three major findings. First, GPs’ weight management policy appeared to be less targeted on primary prevention, neither on the social-environmental factors of overweight and obesity. The result that GPs are less involved in the weight management of people with moderate weight problems is in accordance with a study of Smith et al. [29]. Nonetheless, discussing weight to create awareness at an early stage of weight gain is important as this is the first step in behavioral change [30]. Besides, it is plausible to assert that reaching a healthy weight is easier at an early stage of weight gain. In addition, discussing the influence of medication use, psychosocial problems and environmental factors on patient’s weight management should be encouraged, as these appeared to be talked about less frequently. The importance of these topics is frequently described in the literature. For example, overeating is a common coping mechanism in emotional distress [31]. The environmental availability of healthy or unhealthy food is related to individuals’ food choices [32].

A second major finding of this study is that GPs’ personal characteristics such as age, BMI and concerns appeared to be related to their reported weight management policy. Part of these findings may be explained by the reported negative attitudes towards obese patients among younger GPs [22]. Similar to the findings of Brotons et al.,[33] a relationship between GPs’ BMI and frequency of discussing weight was absent. Possibly, overweight and obese GPs do not believe in an effective treatment of obesity in general. However, this should be studied further. From a patient’s perspective, overweight or obese GPs negatively affect credibility, level of trust and intention to follow weight management advice [34]. Therefore, GPs need to be aware of how they can act as a positive health role model by having a healthy BMI themselves. Finally, GPs who believed that promotion of a healthy weight is an important part of GP care likewise discussed weight more often. This implies that, in order to increase GPs frequency of discussing weight, GPs’ consciousness of weight management as part of their care should be stimulated, recommended by others as well [35]. With respect to this study that investigated the relationship between GPs’ characteristics and their weight management policy, there is a lack of information about the relationship between patients’ characteristics (e.g. age, sex, social economic status) and GPs’ weight management.

A third important finding is that GPs weight management policy can be improved on several ways. Only half of GPs refer their obese patients to other health care providers for dietary treatment and weight is not always discussed, although guidelines recommend doing it. GPs reported several reasons for not talking about weight, with lack of time as the most important cause. This result was in keeping with the findings of other studies [17, 26] and comprehensible with the fact that Dutch GPs are paid per patient by the primary health care insurance cover, based on an average consultation time of 10 minutes [36]. However, by preventing weight-related diseases by means of optimal weight management, it is presumable that by referring to a weight management specialist, GPs could save time in the end.

Next, patients’ lacking motivation was reported as a reason for GPs to not discuss patients’ weight problem, neither refer the patient to a dietitian. But in fact, patients’ lacking motivation is one of the main factors of failing weight management [37]. Therefore, patients’ lacking motivation should be a signal to discuss patients’ weight problem, use motivational interviewing and eventually refer to a specialized caregiver [37].

In contrast to other studies [26], only one third of the respondents reported costs as an important reason for not referring to other health care providers. In the Netherlands, costs may be of little importance due to the system of reimbursement of dietary treatment from Dutch primary health insurance cover. To date, three hours of dietary treatment is included in the standard health insurance package of all Dutch citizens which is obligatory for all Dutch citizens.

Remarkably, the presence of a dietitian in the same medical building was no indication for significantly higher referral rates. Problems in interdisciplinary communication are frequently mentioned in the literature [17, 24, 25]. The present findings indicate that GPs and dietitians should, even when they work in the same building, actively support frequent interdisciplinary communication, for example by providing face-to-face information about their processes [38].

This study has several limitations that may affect interpretation of the results. First, the number of non responders was substantial. In future studies, the response rate might be improved when using incentives, however, for the present study there was no budget available. Since web-based questionnaires appeared to result in higher response-rates [39, 40], this is a recommendation for future studies as well. The second limitation is that the validity and reliability of the questionnaire, though developed carefully, is unclear. Final limitation is the potential information bias. Possibly respondents have provided socially acceptable answers to the questions which may have resulted in a overestimation of the number of GPs with a healthy BMI and the frequency of discussing weight issues with patients. Studies that surveyed patients’ experiences, reported that GPs only intervened in half of the cases with obesity [11, 12]. The contrast between GPs’ reporting and patients’ experiences implicates for future studies that referral percentages need to be confirmed by using data from patient records. Other recommendations for further research are to assess the weight loss of patients referred to a dietitian in comparison to the weight loss of patients without a referral to a dietitian or to another health care provider in weight management.

The key strength of this study is the survey of GPs’ perception of their overweight and obesity management policy. Besides examining GPs’ self-reported frequency of discussing weight as well as their percentages for obesity management, we investigated factors associated with GPs’ weight management policy. The representative population of GPs from all over the Netherlands strengthens the study’s reliability.

In conclusion, this study showed that GPs’ self-reported weight management policy is in accordance with the professional guideline. Nonetheless, in the context of prevention, discussing weight at an early stage of weight gain deserves more attention, especially for younger GPs. Education programs should emphasize the importance of discussing the influence of medication use, psychosocial problems and environmental factors on weight gain. To increase the referral percentage for obesity management, it is important for GPs and dietitians to strengthen interdisciplinary collaboration. Shared feelings of responsibility between GPs and specialists in dietary treatment could play a fundamental role in the struggle to beat overweight and obesity.

## Electronic supplementary material

Additional file 1: Table S1: Questionnaire. **Table S2.** General characteristics from the NIVEL database. (DOCX 19 KB)
