# Supplementary material for: Dutch General Practitioners’ weight management policy for overweight and obese patients
Source: BMC Obes. 2014 Feb 19;1:2. doi: 10.1186/2052-9538-1-2 (PMC4472618; doi:10.1186/2052-9538-1-2)
Supplement: Supplementary file 1 — Additional file 1: Table S1: Questionnaire. Table S2. General characteristics from the NIVEL database. (DOCX 19 KB) [file 40608_2013_2_MOESM1_ESM.docx]

***Box 1. Questionnaire***

| 1 | The presented statements are about your perception of overweight and obesity management. Please indicate to what extent you agree with the next statements? (Scale 1-5, 1=fully disagree, 5=fully agree) |
| --- | --- |
|  | 1. Promoting healthy weight is an important part of GP care |
|  | 1. In my opinion, GPs should educate obese patients (BMI≥30) about potential health risks of their BMI |
|  | 1. In my opinion, GPs should discuss weight with obese patients, even when the patient visits the consultation for another complaint |
| 2 | How often do you talk about weight during consultations, in the following cases: (Scale 1-4, 1=never, 4=always) |
|  | 1. In case of overweight (BMI 25-30) and serious increased waist circumference |
|  | 1. In case of overweight (BMI 25-30) and increased cardiovascular risks |
|  | 1. In case of overweight (BMI 25-30) and weight related comorbidity (for example osteoarthritis, DMII) |
|  | 1. In case of overweight (BMI 25-30), without weight related comorbidity |
|  | 1. In case of obesity (BMI≥30) and increased cardiovascular risks (for example familial CVD, high blood pressure) |
|  | 1. In case of obesity (BMI≥30) and weight related comorbidity (for example osteoarthritis, DMII) |
|  | 1. In case of obesity (BMI≥30), without weight related comorbidity |
| 3 | In case of not talking about weight with an obese patient (BMI≥30) during consultations, what are the reasons for this? (multiple answers allowed) |
|  | 1. Does not apply, I always talk about weight |
|  | 1. I already talked about weight |
|  | 1. I do not have enough time |
|  | 1. I do not know what kind of advice I should give |
|  | 1. I am scared to affect the relation with the patient |
|  | 1. I am overweight as well, because of that I might be implausible at this point |
|  | 1. I believe, talking about weight is not GPs duty of care |
|  | 1. I believe, the patient should start the conversation about weight |
|  | 1. I believe, education does not work in case of obesity |
|  | 1. I believe, talking about weight is not meaningful if the patient has a healthy lifestyle |
|  | 1. I believe, talking about weight is not meaningful if the patient is demotivated |
| 4 | In case of talking about weight with an obese patient (BMI≥30) during consultations, which subjects are usually discussed? (multiple options possible) |
|  | 1. Does not apply, I never talk about weight |
|  | 1. Patients’ motivation for weight loss |
|  | 1. The environmental influences (for example family or type of job) on weight |
|  | 1. Patients vision about a good and healthy weight |
|  | 1. Weight loss attempts in the past |
|  | 1. Patients’ current level of physical activity |
|  | 1. Patients’ current diet |
|  | 1. Patients’ current medication use |
|  | 1. Psychosocial problems (for example a negative self-image) |
|  | 1. Realistic targets for weight loss |
|  | 1. Weight related health risks |
|  | 1. The possibilities for weight loss |
| 5 | To what extent do you think the next caregivers are suitable for dietary treatment of obese patients? GP, GPs nurse practitioner, dietitian, weight consultant, psychologist, physical therapist (Scale 1-4, 1=not at all, 4=very suitable) |
| 6 | Are you frequently in contact with a dietitian? (0=No, 1=Yes) |
| 7 | Are there caregivers offering nutrition and/or dietary advisement in your medical center? Me (GP), practical nurse, dietitian, weight consultant, nurse, psychologist, physical therapist. Multiple options are possible. |
| 8 | What percentage of obese people who visit your consultation, do you refer to a dietitian for nutrition- and/or dietary advice? Give an estimation between 0 of to 100% |
| 9 | In case an obese patient, what kind of scenario is fitting with patterns in your treatment? Most of the time…(0=No, 1=yes) |
|  | 1. I immediately refer to another caregiver |
|  | 1. My advice is to lose weight non-supervised. If this did not work, I start a treatment by myself |
|  | 1. My advice is to lose weight non-supervised. If this did not work, I start a treatment by myself. If this has failed, I refer to another caregiver |
|  | 1. My advice is to lose weight non-supervised. If this did not work, I refer to another caregiver |
|  | 1. I start a treatment by myself. If this did not work, I refer to another caregiver |
| 10 | In case of not referring obese patients to a dietitian, what are the most important reasons for this? |
|  | 1. Self-management is enough |
|  | 1. Other weight loss methods are more effective (like a diet-book or surgery) |
|  | 1. Dietitians’ dietary treatment is not effective |
|  | 1. Other caregivers are more effective |
|  | 1. Dietary-costs are too high |
|  | 1. In my region, I do not know a dietitian to recommend |
|  | 1. In my region, I do not know a dietitian who delivers a combined lifestyle intervention. |
|  | 1. Patients do not want a dietetic-treatment |
|  | 1. Patients do not have enough motivation for dietary support |
|  | 1. My own treatment is better than other treatments |
| 11 | What is your height, measured in centimeters? |
| 12 | What is your current body weight, measured in kilogram? If you are pregnant, mention the pre-pregnancy weight |

***Box 2. General characteristics from the NIVEL database***

| 1 | Gender (1=man, 2=woman) |
| --- | --- |
| 2 | Age at January 1^st^, 2013 (continuous) |
| 3 | Type of employment (1= private, 2=salaried) |
| 4 | Type of practice (1= solo practice, 2= duo practice, 3= group practice) |
| 5 | Urbanicity (Scale 1-5, 1=urban, 5=rural)* |

*Urbanicity: 1) Urban: >2500 addresses per km^2^. 2) Urban to Suburban: 1500-2499 addresses per km^2^. 3)Suburban: 1000-1499 addresses per km^2^. 4)Suburban to rural: 500-9999 addresses per km^2^. 5)Rural: <500 addresses per km^2^.
